# Supplementary material for: BZLF1 interacts with chromatin remodelers promoting escape from latent infections with EBV
Source: Life Sci Alliance. 2019 Mar 29;2(2):e201800108. doi: 10.26508/lsa.201800108 (PMC6441497; doi:10.26508/lsa.201800108)
Supplement: Supplementary file 1 [file LSA-2018-00108_Supplemental_Data_1.docx]

**BZLF1 interacts with chromatin remodelers promoting escape from latent infections with Epstein-Barr virus**

SUPPLEMENTARY MATERIAL

Marisa Schaeffner^1,2†^, Paulina Mrozek-Gorska^1,2†^, Alexander Buschle^1,2^, Anne Woellmer^1,2^, Takanobu Tagawa^1,2^, Filippo M. Cernilogar^3^, Gunnar Schotta^3,4^, Nils Krietenstein^3^, Corinna Lieleg^3^, Philipp Korber^3^, and Wolfgang Hammerschmidt^1,2*^

^1^Research Unit Gene Vectors

Helmholtz Zentrum München

German Research Center for Environmental Health

Marchioninistr. 25

D-81377 Munich, Germany

^2^German Center for Infection Research (DZIF), Partner site Munich, Germany

^3^Biomedical Center, Molecular Biology, LMU Munich, 82152 Planegg-Martinsried, Germany

^4^Center for Integrated Protein Science Munich

^†^equal author contributions

*Corresponding author

Wolfgang Hammerschmidt

Phone: +49-89-3187-1506

Fax: +49-89-3187-4225

E-mail: [hammerschmidt@helmholtz-muenchen.de](mailto:hammerschmidt@helmholtz-muenchen.de)

MATERIALS AND METHODS

**Chromatin immunoprecipitation (ChIP) and sequential chromatin immunoprecipitation (ReChIP)**

ReChIP experiments with the anti-BZLF1 antibody as first antibody started with 1x10^8^ Raji p4816 cells, which were washed twice with ice-cold PBS and cross-linked in 40 ml PBS with a final concentration of 1 % formaldehyde at room temperature with constant rotating for 8 minutes. Cross-linking was quenched with 5 ml 1 M glycine for 1 min at room temperature followed by incubation on ice for 5 minutes. Cells were again pelleted and washed twice with ice-cold PBS. For nuclei preparation the pellet was washed with 10 ml of ReChIP lysis buffer 1 (50 mM HEPES-KOH pH 7.5, 140 mM NaCl, 1 mM EDTA pH 8.0, 0.5 mM EGTA pH 8.0, 10 % glycerol, 0.5 % Igepal, 0.25 % Triton X-100) and ReChIP lysis buffer 2 (25 mM HEPES pH 7.5, 200 mM NaCl, 1 mM EDTA pH 8.0, 0.5 mM EGTA pH 8.0), resuspended in 5 ml ReChIP lysis buffer 3 (25 mM HEPES pH 7.5, 140 mM NaCl, 1 mM EDTA pH 8.0, 0.5 mM EGTA pH 8.0, 0.25 % N-lauryl sarcosine, 0.1 % sodium deoxycholate, 0.25 % Triton X-100) and the occurrence of grey colored nuclei was confirmed under the microscope. To obtain mononucleosome-sized chromatin fragments of 150 bp, nuclei were sonicated (Branson sonifier W-250 D, amplitude 35 %, total pulse time 240 s with 120 cycles of 1 s pulse and 1 s pause) in the presence of acid-washed glass beads (212 to 300 µm, Merck). Sonicated samples were centrifuged and further digested with 10 U/µl Micrococcal Nuclease S7 (Roche) in a final concentration of 4 mM CaCl_2_ for 10 minutes at 37 °C. The digest was stopped by EGTA pH 8.0 at a final concentration of 40 mM. The DNA content and the size of sheared and digested chromatin was estimated by Nanodrop measurement (Thermo Scientific) and agarose gel electrophoresis, respectively, and the chromatin solution was adjusted to 1 mg DNA per ml with final concentrations of 0.5 % Triton X-100, 10 % glycerol in ReChIP lysis buffer 3.

1500 µg of chromatin was adjusted with ReChIP lysis buffer 3 to a final volume of 5 ml. 25 µg of the BZLF1 antibody was added and incubated over night with constant overhead rotating. 500 μl of protein G resin (50 % slurry) were pre-equilibrated with 4.5 ml ReChIP lysis buffer 3 and incubated over night while rotating. The antibody-chromatin mixture was added to the pelleted beads and mixed for 2 hours by overhead rotating. The beads were washed in 1 ml ReChIP wash buffer 1 (50 mM Tris-HCl, pH 8.0, 150 mM NaCl, 1 mM EDTA, pH 8.0, 0.1 % SDS, 0.5 % sodium deoxycholate, 1 % Igepal), ReChIP wash buffer 2 (50 mM Tris-HCl, pH 8.0, 300 mM NaCl, 1 mM EDTA, pH 8.0, 0.1 % SDS, 0.5 % sodium deoxycholate, 1 % Igepal), ReChIP LiCl buffer (50 mM Tris-HCl, pH 8.0, 250 mM LiCl, 0.1 % SDS, 0.5 % sodium deoxycholate, 1 % Igepal) and TE buffer (10 mM Tris-HCl, pH 8.0, 1 mM EDTA). Elution of the BZLF1-ChIPed chromatin was performed with 100 µl ChIP sonication buffer (50 mM Tris-HCl pH 7.5, 10 mM EDTA, 1 % SDS, 1 % Igepal) by incubating for two hours at 37 °C under constant shaking. For the second round of ChIP, the first ChIPed chromatin solution was adjusted with ChIP sonication buffer to a final volume of 1 ml and a final concentration of 0.1 % SDS. 1 µg of H3K4me1 antibody (Abcam, #8895) was added and incubated at 4 °C overnight with constant overhead rotating. 100 μl of protein A resin (50 % slurry) were pre-equilibrated with 1 ml ChIP sonication buffer and incubated at 4 °C overnight with constant overhead rotating. The next day, beads were spun down, added to the antibody-chromatin mixture, and incubated at 4 °C for two hours by overhead rotating. To remove unspecific DNA and proteins, the beads were washed with 1 ml of ChIP low salt buffer (20 mM Tris-HCl, pH 8.0, 2 mM EDTA, pH 8.0, 1 % Triton X-100, 150 mM NaCl, 0.1 % SDS), ChIP high salt buffer (20 mM Tris-HCl, pH 8.0, 2 mM EDTA, pH 8.0, 1 % Triton X-100, 500 mM NaCl, 0.1 % SDS), ChIP LiCl buffer (10 mM Tris-HCl, pH 8.0, 1 mM EDTA, pH 8.0, 250 mM LiCl, 0.5 % Igepal, 0.5 % sodium deoxycholate) and TE buffer (10 mM Tris-HCl, pH 8.0, 1 mM EDTA). Elution and removal of the cross-link was accomplished in 200 µl ChIP elution buffer (25 mM Tris-HCl, pH 7.5, 10 mM EDTA, pH 8.0, 0.5 % SDS) containing 20 µg proteinase K for three hours at 68 °C while shaking.

ReChIP experiments in reverse order starting with an anti-H3K4me1 antibody (Abcam, #8895) were performed as follows. For the first round of ChIP 100 µg of chromatin solution (prepared according to Woellmer et al., 2012) was adjusted with ChIP sonication buffer to a final volume of 1 ml and a final concentration of 0.1 % SDS. 5 µg of H3K4me1 antibody was added and incubated at 4 °C overnight with constant overhead rotating. 100 μl of protein A resin (50 % slurry) were pre-equilibrated with 1 ml ChIP dilution buffer and incubated at 4 °C overnight with constant overhead rotating. The next day, beads were spun down, added to the antibody-chromatin mixture, and incubated at 4 °C for two hours by overhead rotating. To remove unspecific DNA and proteins, the beads were washed with 1 ml of ChIP low salt buffer, ChIP high salt buffer, ChIP LiCl buffer and TE buffer. Elution of the H3K4me1-ChIPed chromatin was accomplished with 100 µl ReChIP lysis buffer 3 and 1 % SDS by incubating for two hours at 37 °C under constant shaking. For the second round of ChIP, the first H3K4me1-ChIPed chromatin solution was adjusted with ReChIP buffer 3 to a final volume of 1 ml and a final concentration of 0.1 % SDS. 1 µg of a BZLF1 antibody (Santa Cruz, #17503) was added and incubated at 4 °C overnight with constant overhead rotating. 15 μl of protein G resin (50 % slurry) were pre-equilibrated with 1 ml ReChIP buffer 3 and incubated at 4 °C overnight with constant overhead rotating. The next day, beads were spun down, added to the antibody-chromatin mixture, and incubated at 4 °C for two hours by overhead rotating. To remove unspecific DNA and proteins, the beads were washed with 1 ml of ReChIP wash buffer 1, ReChIP wash buffer 2, ReChIP LiCl buffer and TE buffer. Elution and removal of the cross-link was accomplished in 200 µl ChIP elution buffer containing 20 µg proteinase K for three hours at 68 °C while shaking.

**INO80 ChIP and qPCR**

6x10^6^ Raji p4816 cells were induced with 100 ng/ml doxycycline for 6 and 15 hours. The non-induced (0h) and induced cells were controlled by FACS for GFP expression, collected by centrifugation (300 g, 10 min) and washed twice with PBS. The cell pellets were used to extract chromatin and for immunoprecipitation with the ChIP kit (Abcam #ab500) according to the manufacturer’s recommendations. Briefly, cells were fixed for 12 min with 1% formaldehyde, the reaction was quenched with glycine and the cells were lysed on ice. The chromatin was sheared to fragments of 200-300 bp in length using a Covaris S220 ultra-sonicator. Chromatin from approximately 1x10^6^ cells was incubated with 5 µg of the INO80 antibody (Proteintech #188010-1-AP) at 4°C overnight. The next day the chromatin/antibody solution was mixed and incubated with prepared protein A Sepharose beads for 2 h at 4°C. In the next step, the beads were pelleted and washed with 1xChIP buffer (ChIP kit, Abcam #ab500) four times followed by de-cross-linking and DNA extraction. ChIP DNA samples were analyzed by qPCR using the primers listed in Table S2 with the aid of a LightCycler 480 instrument (Roche). Values representing percent of the input, their mean and standard deviations were calculated from four independent biological replicates.

**Generation of Raji p4816 cell lines with lentiviral shRNA vectors directed against INO80**

Lentiviruses were produced by co-transfecting lentiviral vector DNA and additional plasmids required for lentiviral packaging in 293T cells. Transfections were done with Turbofect. 1x10^7^ 293T cells were seeded one day prior to transfection. The next day medium was changed to fresh DMEM medium. Cells were transfected with a cocktail of 7 µg pCMV-dR8.91 plasmid encoding the *gag*, *pol* and *rev* genes (p4502) (Zufferey et al., 1997); 8 µg pMD2.G (p5451) encoding the VSV-G expressing envelope plasmid; 6 µg of an expression plasmid encoding gp350 (p2385); and 11 µg pCDH vector DNA with the shRNA sequences (p6574, p6577, p6578). After 72 hours supernatants from the transfected plates were collected, centrifuged with 300 g for 10 min and cleared of debris using 0.45 µm pore size membrane filters. Lentiviral titers in the supernatants were estimated by infecting Raji or THP1 cells and recording DsRed expression by flow cytometry after five to seven days.

For transduction of Raji p4816 cells, they were seeded in 24-well cluster plates at 5x10^5^ cells/well in 700 µl cell culture medium and protamine sulfate was added to a final concentration of 10 µg/ml. 300 µl of virus supernatant was added, mixed and centrifuged at 800 g at 32°C for 50 min. The cells were transferred to an incubator and medium containing puromycin was added the next day. Cells were expanded and DsRed positive cells were enriched by physical sorting using a FACSAria IIIu cell sorter (Becton Dickinson). DsRed-positive cells were collected, expanded further and cell lysates were obtained for the analysis of INO80 protein levels by immunoblotting.

**Quantification of transcripts by RT-qPCR**

Raji p4816 cells which express lentivirally transduced shRNA_nt or two INO80 targeting shRNAs (shRNA_INO80_1 and _2) were induced for BZLF1 expression (100 ng/ml doxycycline) for 2, 4, 8, or 15 hours. From non-induced and from induced cells fractions were collected at the indicated time points to determine the induced expression of GFP by flow cytometry and to analyze the INO80 protein levels by immunoblot detection. At each time point 1×10^6^ cells were centrifuged, washed with ice cold PBS, and pellets were snap frozen in -80 °C. Isolation of cellular RNA was accomplished with the RNeasy Mini Kit (Qiagen). The cell lysates were homogenized with QiaShredder columns (Qiagen). The subsequent steps were performed according to the manufacturer’s instructions. RNA was eluted in 30 µl of RNase-free water. Contaminating genomic DNAs were removed with RNAse-free deoxyribonuclease I (DNase I, Thermo Fisher Scientific) in the presence of RNase inhibitor (Roche) at room temperature for 40 min followed by inactivation at 65 °C for 10 min. The efficiency of DNA digestion was determined by PCR amplification of viral DNA with the BBLF2 primer pair (Tab. S2). RNA was reverse-transcribed into cDNA using the SuperScript III First Strand Synthesis SuperMix Kit (Thermo Fisher Scientific) following the manufacturer’s instructions. cDNA was analyzed quantitatively by RT-qPCR using primers listed in Table S2 and a LightCycler480 instrument (Roche).

**Next generation BZLF1 ChIP-sequencing**

For each sample 1x10^8^ Raji p4816 cells were adjusted to a final concentration of 5x10^5^ cells/ml in fresh medium. Cells were non-induced or induced for 15 h with a final concentration of 100 ng/ml doxycycline (Sigma-Aldrich). Hypotonic buffer (10 mM KCl, 340 mM Sucrose, 1.5 mM MgCl_2_, 10 mM Hepes pH 7.9) containing 10 % proteinase inhibitor cocktail (PIC, Roche) was used to extract nuclei, which were subsequently lysed in RIPA buffer + 1x PIC. A BioRuptor was used to share chromatin on wet ice (4 cycles, 5 min each, 30 sec on/off, high) before immunoprecipitation with the BZ1 antibody (Young et al., 1991). While 10 % input was used as a control, the precipitates were washed with different salt concentrations and the protein-DNA aggregates were digested with proteinase K. The library preparations and sequencing (Illumina, paired-end, 150 nt) were performed by Vertis Biotechnology AG (Freising, Germany). Bowtie2 (Langmead and Salzberg, 2012) was used for mapping, samtools (Li et al., 2009) and bedtools (Quinlan and Hall, 2010) were used for format transformations, and MACS2 (Feng et al., 2012; Zhang et al., 2008) for peak calling of the samples versus input. Data sets are listed in Tab. S3.

**Next generation CTCF ChIP-sequencing**

Chromatin preparation and immunoprecipitation with the anti-CTCF antibody (Upstate, #07-729) were carried out essentially as described in the previous paragraph. Illumina next generation sequencing was done in cooperation with Vertis Biotechnology AG (Freising). Input and ChIP samples were end-repaired and ligated with TruSeq adapters. Libraries were PCR-amplified to about 50 ng/µl using a high-fidelity DNA polymerase, if necessary. Barcoded samples were pooled in equimolar amounts and DNA in the range of 300-500bp was eluted from preparative agarose gels. The pools were sequenced on an Illumina HiSeq 2000 instrument with single reads of 100 bases. ChIP-seq reads were aligned to a joint genome comprising human (version hg19) and the Raji sequences (accession number KF717093) using bowtie 0.12.8 (Langmead et al., 2009) omitting hits mapping to multiple genomic locations. For calculation of coverage vectors after ChIP-seq experiments we extended each read to 200 nucleotides. MACS2 (Yang et al., 2014) was applied for calling peaks on mapped target reads using chromatin input reads as control with the parameters “–-nomodel” and “--extsize 200”. The data sets are listed in Tab. S3.

**ATAC-seq analysis**

1x10^6^ Raji cells stably transfected with either the inducible wild-type BZLF1 allele (p4816) or the AD-truncated BZLF1 allele (p5694) were induced with 100 ng/ml doxycycline for 15 h or kept untreated. The cells were FACS sorted for living cells according to forward and sideward scatter criteria and doxycycline-induced cells were sorted for GFP-positivity to enrich for induced cells. Omni-ATAC-seq was performed as previously described (Corces et al., 2017) with minor modifications. Briefly, 50,000 FACS sorted cells were washed in PBS, resuspended in 50 μl of ATAC-seq resuspension buffer (RSB: 10 mM Tris-HCl, pH 7.4, 10 mM NaCl, and 3 mM MgCl_2_) containing 0.1 % NP40, 0.1 % Tween-20 and 0.01 % digitonin (Promega) and were incubated on ice for 3 min. Following lysis, 1 ml of ATAC-seq RSB containing 0.1 % Tween-20 was added, and the nuclei were centrifuged at 500 g (4 °C, 10 min). The pelleted nuclei were resuspended in 50 μl transposition mix (25 μl 2 × TD buffer, 2.5 μl Tagment DNA enzyme [Illumina Nextera DNA Library Preparation Kit, cat. FC-121-1030], 16.5 μl PBS, 0.5 μl 1 % digitonin, 0.5 μl 10 % Tween-20, and 5.25 μl water) and incubated at 37 °C for 30 min in a thermomixer shaking at 1,000 rpm. The DNA was purified using Qiagen PCR clean-up MinElute kit (Qiagen). The transposed DNA was subsequently amplified in 50 μl reactions with custom primers as described (Buenrostro et al., 2013). After four PCR cycles, the libraries were monitored by qPCR in a 15 μl reaction volume with 5 μl of PCR sample using the previous PCR primer pair. The qPCR output was monitored for the ΔRN; 0.25 ΔRN cycle number was used to estimate the number of additional cycles of the PCR reaction needed for the remaining PCR samples. Amplified libraries were purified with the Qiagen PCR clean-up MinElute kit (Qiagen) and size selected for fragments <600 bp using Agencourt AMPure XP beads (Beckman Coulter). Libraries were quality controlled by Qubit and Agilent DNA Bioanalyzer analyses. High throughput sequencing was performed by the Laboratory for Functional Genome Analysis (LAFUGA) of the Ludwig-Maximilian-University, Munich, on an Illumina Hi-Seq 1500 using 50 nt single-end reads.

The data were mapped on the Raji genome KF717093.1 with Bowtie 1.1.2. The HOMER software (version 4.9) was used to calculate tag directories. The data for the metaplots, boxplots and heatmaps were calculated with HOMER’s annotate Peaks.pl and visualized with R (ver. 3.5.1) (Team, 2018)[. For heatmaps visualization the data.ta](https://www.r-project.org/\)ble (https://cran.r-project.org/web/packages/data.table/index.html) and superheat package (Barter and Yu, 2017) were applied. For the visualization of the ATAC-seq data on the Raji genome KF717093.1 the IGV browser was used (Robinson et al., 2011; Thorvaldsdóttir et al., 2013). The data sets are listed in Tab. S3.

**References of Supplementary Material**

Barter RL, Yu B (2017) Superheat: An R package for creating beautiful and extendable heatmaps for visualizing complex data. 1–26

Buenrostro JD, Giresi PG, Zaba LC, Chang HY, Greenleaf WJ (2013) Transposition of native chromatin for fast and sensitive epigenomic profiling of open chromatin, DNA-binding proteins and nucleosome position. Nat Methods 10:1213–1218. doi: 10.1038/nmeth.2688

Corces MR, Trevino AE, Hamilton EG, Greenside PG, Sinnott-Armstrong NA, Vesuna S, Satpathy AT, Rubin AJ, Montine KS, Wu B, Kathiria A, Cho SW, Mumbach MR, Carter AC, Kasowski M, Orloff LA, Risca VI, Kundaje A, Khavari PA, Montine TJ, Greenleaf WJ, Chang HY (2017) An improved ATAC-seq protocol reduces background and enables interrogation of frozen tissues. Nat Methods 14:959–962. doi: 10.1038/nmeth.4396

Delecluse HJ, Hilsendegen T, Pich D, Zeidler R, Hammerschmidt W (1998) Propagation and recovery of intact, infectious Epstein-Barr virus from prokaryotic to human cells. Proc Natl Acad Sci U S A 95:8245–8250. doi: 10.1073/pnas.95.14.8245

Feng J, Liu T, Qin B, Zhang Y, Liu XS (2012) Identifying ChIP-seq enrichment using MACS. Nat Protoc 7:1728–1740. doi: 10.1038/nprot.2012.101

Krietenstein N, Wippo CJ, Lieleg C, Korber P (2012) Genome-wide in vitro reconstitution of yeast chromatin with in vivo-like nucleosome positioning. Methods Enzymol 513:205–232. doi: 10.1016/B978-0-12-391938-0.00009-4

Langmead B, Salzberg SL (2012) Fast gapped-read alignment with Bowtie 2. Nat Methods 9:357–359. doi: 10.1038/nmeth.1923

Langmead B, Trapnell C, Pop M, Salzberg SL (2009) Ultrafast and memory-efficient alignment of short DNA sequences to the human genome. Genome Biol 10:R25. doi: 10.1186/gb-2009-10-3-r25

Li H, Handsaker B, Wysoker A, Fennell T, Ruan J, Homer N, Marth G, Abecasis G, Durbin R, 1000 GPDPS (2009) The Sequence Alignment/Map format and SAMtools. Bioinformatics 25:2078–2079. doi: 10.1093/bioinformatics/btp352

Lowary PT, Widom J (1998) New DNA sequence rules for high affinity binding to histone octamer and sequence-directed nucleosome positioning. J Mol Biol 276:19–42. doi: 10.1006/jmbi.1997.1494

Quinlan AR, Hall IM (2010) BEDTools: a flexible suite of utilities for comparing genomic features. Bioinformatics 26:841–842. doi: 10.1093/bioinformatics/btq033

Team RC (2018) R: A language and environment for statistical computing.

Robinson JT, Thorvaldsdóttir H, Winckler W, Guttman M, Lander ES, Getz G, Mesirov JP (2011) Integrative genomics viewer. Nat Biotechnol 29:24–26. doi: 10.1038/nbt.1754

Thorvaldsdóttir H, Robinson JT, Mesirov JP (2013) Integrative Genomics Viewer (IGV): high-performance genomics data visualization and exploration. Brief Bioinform 14:178–192. doi: 10.1093/bib/bbs017

Watanabe C, Cuellar TL, Haley B (2016) Quantitative evaluation of first, second, and third generation hairpin systems reveals the limit of mammalian vector-based RNAi. RNA Biol 13:25–33. doi: 10.1080/15476286.2015.1128062

Woellmer A, Arteaga-Salas JM, Hammerschmidt W (2012) BZLF1 governs CpG-methylated chromatin of Epstein-Barr virus reversing epigenetic repression. PLoS Pathog 8:e1002902. doi: 10.1371/journal.ppat.1002902

Yang Y, Fear J, Hu J, Haecker I, Zhou L, Renne R, Bloom D, McIntyre LM (2014) Leveraging biological replicates to improve analysis in ChIP-seq experiments. Comput Struct Biotechnol J 9:e201401002

Young LS, Lau R, Rowe M, Niedobitek G, Packham G, Shanahan F, Rowe DT, Greenspan D, Greenspan JS, Rickinson AB, et A (1991) Differentiation-associated expression of the Epstein-Barr virus BZLF1 transactivator protein in oral hairy leukoplakia. J Virol 65:2868–2874

Zhang Y, Liu T, Meyer CA, Eeckhoute J, Johnson DS, Bernstein BE, Nusbaum C, Myers RM, Brown M, Li W, Liu XS (2008) Model-based analysis of ChIP-Seq (MACS). Genome Biol 9:R137. doi: 10.1186/gb-2008-9-9-r137

Zufferey R, Nagy D, Mandel RJ, Naldini L, Trono D (1997) Multiply attenuated lentiviral vector achieves efficient gene delivery in vivo. Nat Biotechnol 15:871–875. doi: 10.1038/nbt0997-871
