# Supplementary material for: BZLF1 interacts with chromatin remodelers promoting escape from latent infections with EBV
Source: Life Sci Alliance. 2019 Mar 29;2(2):e201800108. doi: 10.26508/lsa.201800108 (PMC6441497; doi:10.26508/lsa.201800108)
Supplement: Supplementary file 2 [file LSA-2018-00108_TableS1.doc]

Table S1. qPCR program for Roche LightCycler 480 instrument

| Program | Target temperature (°C) | Hold (sec) | Acquisition mode | RampRate (°C/sec) | Cycles | Analysis mode |
| --- | --- | --- | --- | --- | --- | --- |
| Pre-incubation | 95 | 600 | None | 4.4 | 1 | None |
| Amplification | 95  62  72  75 | 1  10  10  3 | None  None  None  Single | 4.4  2.2  4.4  4.4 | 45 | Quantification |
| Melting curve | 97 | 1 | None | 4.4 | 1 | Melting curve |
|  | 67 | 10 | None | 2.2 |  |  |
|  | 97 | / | Continuous | 0.11 |  |  |
| Cooling | 37 | 15 | None | 2.2 | 1 | None |
