# Supplementary material for: BZLF1 interacts with chromatin remodelers promoting escape from latent infections with EBV
Source: Life Sci Alliance. 2019 Mar 29;2(2):e201800108. doi: 10.26508/lsa.201800108 (PMC6441497; doi:10.26508/lsa.201800108)
Supplement: Supplementary file 3 [file LSA-2018-00108_TableS2.doc]

**Table** S2 Oligonucleotides for qPCR analyses

| **Locus** | **Forward Primer** | **Reverse Primer** |
| --- | --- | --- |
| *cen* | AAGGTCAATGGCAGAAAAGGA | CAACGAAGGCCACAAGATGTC |
| *GAPDH* | CCCCGGTTTCTATAAATTGAGC | GGCTGACTGTCGAACAGGA |
| *HPRT1* | CTGAGTTGGGAGGGAAAGG | CACTAGGTAGCCGTGGGAAT |
| *TAP2* | GAATGGCCGGAAGAGGGAAG | AGAAGCTAGGGTCTGGAGGG |
| *SLCO2B1* | TTTGCCCACAACAGCAACTC | TGGTTAATGTCCACATAAAGGCG |
| *HOXB13* | ACACAGGCGTCTGTATTC | ACACAGGCGTCTGTATTC |
|  |  |  |
| EBER | CGCTACATCAAACAGGACAGC | AGCCGAATACCCTTCTCCCAG |
| C | ACAAGGGGACAAGTGTGGCA | TGACTGGTGGGGGGCATC |
| Q/F | TGTCACCACCTCCCTGATAATGTC | CATACACCGTGCGAAAAGAAGC |
| W | GCCTAAAACCCCCAGGAAGC | GACCCCCTCTTACATTTGTGT |
| BRLF1 | CCGGCTGACATGGATTACTGG | AGGAACCAAAATAACCGAGCCTC |
| BZLF1 | GGTGCAATGTTTAGTGAGTTACCTGTC | TGACACCAGCTTATTTTAGACACTTCTG |
| BMRF1 | CACACCACCCCCCAAGGAC | GCAGCAGCAGAAGCCAACG |
| BNLF2a | TGCTGACGTCTGGGTCCT | TGCTTTGCTAGAGCAGCAGT |
| BBLF4 | GAGGCAGGTGTTTACCCATTTG | CAGGTCACGCACGGTCAGC |
| BSRF1 | CCAAAAATAGTAAGCAGCCGTGA | GAAACAGCCACAGGGGGATG |
| BcLF1 | AATCAAATGGTTGGACACGGC | TCAGGGTGGGCAGAGGACC |
| BBLF2 | GTCGGGAGTCTCGGTGGAATAG | AGCACAGGTGGTCTGCCAAAG |
