# Supplementary material for: BZLF1 interacts with chromatin remodelers promoting escape from latent infections with EBV
Source: Life Sci Alliance. 2019 Mar 29;2(2):e201800108. doi: 10.26508/lsa.201800108 (PMC6441497; doi:10.26508/lsa.201800108)
Supplement: Supplementary file 4 [file LSA-2018-00108_TableS3.doc]

**Table S3. Summary table of NGS data in public database**

| Samples Sets | ATAC-seq | Replicate | Database accession ^a^ |
| --- | --- | --- | --- |
| 1 | BZLF1 full-length non-induced | 1 | E-MTAB-7789 |
| 1 | BZLF1 full-length non-induced | 2 | E-MTAB-7789 |
| 1 | BZLF1 full-length non-induced | 3 | E-MTAB-7789 |
| 2 | BZLF1 full-length induced | 1 | E-MTAB-7789 |
| 2 | BZLF1 full-length induced | 2 | E-MTAB-7789 |
| 2 | BZLF1 full-length induced | 3 | E-MTAB-7789 |
| 3 | BZLF1 truncated non-induced | 1 | E-MTAB-7789 |
| 3 | BZLF1 truncated non-induced | 2 | E-MTAB-7789 |
| 3 | BZLF1 truncated non-induced | 3 | E-MTAB-7789 |
| 4 | BZLF1 truncated induced | 1 | E-MTAB-7789 |
| 4 | BZLF1 truncated induced | 2 | E-MTAB-7789 |
| 4 | BZLF1 truncated induced | 3 | E-MTAB-7789 |
|  |  |  |  |
| Samples Sets | ChIP-seq |  |  |
| 1 | BZLF1 full-length non-induced | 1 | E-MTAB-7788 |
| 1 | BZLF1 full-length non-induced | 2 | E-MTAB-7788 |
| 2 | BZLF1 full-length non-induced 10 % input | 1 | E-MTAB-7788 |
| 2 | BZLF1 full-length non-induced 10 % input | 2 | E-MTAB-7788 |
| 3 | BZLF1 full-length induced | 1 | E-MTAB-7788 |
| 3 | BZLF1 full-length induced | 2 | E-MTAB-7788 |
| 4 | BZLF1 full-length induced 10 % input | 1 | E-MTAB-7788 |
| 4 | BZLF1 full-length induced 10 % input | 2 | E-MTAB-7788 |
| 5 | CTCF induced Raji 4816 | 1 | E-MTAB-7787 |
| 6 | CTCF induced Raji 4816 cells 10 % input | 1 | E-MTAB-7787 |

^a^https://www.ebi.ac.uk/arrayexpress/
